# Treatment of an Unusual Occurrence of a Complex Left Subclavian Artery/Left Internal Mammary Artery Bifurcation Stenosis in the Setting of Coronary Subclavian Steal Syndrome and Ischemic Left Ventricular Systolic Dysfunction

**DOI:** 10.1155/2018/9340183

**Published:** 2018-04-23

**Authors:** Michael J. Martinelli, Michael B. Martinelli

**Affiliations:** ^1^St. Peter's Health Partners, 2 Palisades Drive, Albany, NY 12205, USA; ^2^College of Biomedical Sciences, Thomas Jefferson University, Philadelphia, PA, USA

## Abstract

This case will illustrate the clinical and unique technical challenges, not previously reported, in a patient with a history of progressive left ventricular (LV) systolic dysfunction, congestive heart failure (CHF), myocardial infarction (MI), and a complex bifurcation lesion of the left subclavian artery (SA) involving the left internal mammary artery (LIMA) in the setting of coronary subclavian steal syndrome (CSSS). The approach to this lesion is complicated by significant LIMA involvement requiring intervention directed toward both the SA and the LIMA in the presence of severe LV systolic dysfunction. This clinical scenario necessitates a careful technique, utilizing bifurcation methods similar to those used in coronary intervention.

## 1. Introduction

Coronary subclavian steal syndrome (CSSS), first described in 1974, is characterized by a stenotic proximal left subclavian artery (SA) causing reversal of flow in the LIMA resulting in a “steal” phenomenon [1]. This syndrome typically presents with angina during left upper extremity exertion. Less common manifestations include acute myocardial infarction (MI), ischemic cardiomyopathy, and ventricular arrhythmias [2, 3]. The case reported here demonstrates a lesion of the proximal SA, which could be expected to result in CSSS, but which atypically involves both the LIMA and the SA beyond the LIMA. This lesion morphology not only creates a steal phenomenon but further compromises LIMA flow to the LAD by inhibiting inflow to and outflow from the LIMA. This complex anatomy may further worsen the clinical severity beyond which is typically seen in CSSS alone. Both the lesion morphology and the severity of the clinical manifestations noted in this patient increase the risk and technical difficulty of the procedure.

## 2. Case

A 75-year-old Caucasian male was referred for evaluation with the following history: coronary artery bypass grafting (CABG) with left internal mammary artery (LIMA) to left anterior descending (LAD) and sequential saphenous vein grafting (SVG) to ramus, obtuse marginal, right posterolateral and right posterior descending arteries, repeat CABG two years later with revision of the SVG, severe peripheral arterial disease (PAD) with a history of left lower extremity surgical revascularization, right iliac artery occlusion, atherosclerotic carotid artery disease, and chronic kidney disease (CKD) Stage III. He had been hospitalized for the treatment of CHF and non-ST elevation myocardial infarction (NSTEMI) within the prior three months and continued to experience increasing angina and dyspnea on exertion (NYHA Class III), which was refractory to medications. He was noted to have had a decrease in LV ejection fraction by echocardiogram from 45% to 15% and underwent cardiac catheterization.

**Figure 1:**
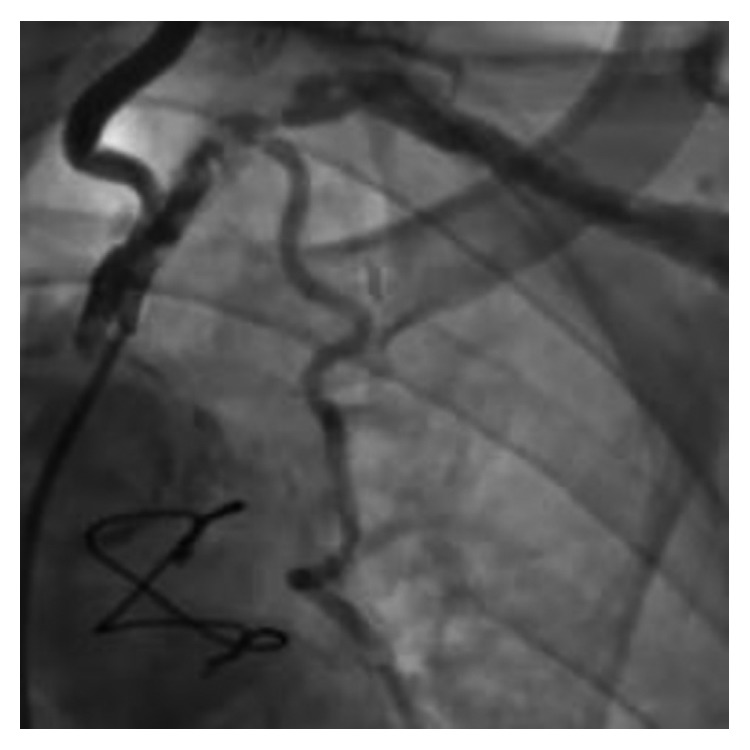
SA angiogram showing diffuse, severe atherosclerosis of the SA/LIMA bifurcation. SA, subclavian artery; LIMA, left internal mammary artery.

Cardiac catheterization revealed a patent sequential SVG and severe disease of the body of the SA, distal to the left vertebral artery, and involving the LIMA (Figure 1). The lesion involved the LIMA and the SA, immediately both proximal and distal to the LIMA. Cardiac surgical consultation was obtained, and the patient was deemed a prohibitive risk for a third CABG. Vascular surgical treatment (subclavian-carotid transposition or bypass) was not an option as it would not address the compromised LIMA. Percutaneous therapy directed toward the SA/LIMA bifurcation was planned.

**Figure 2:**
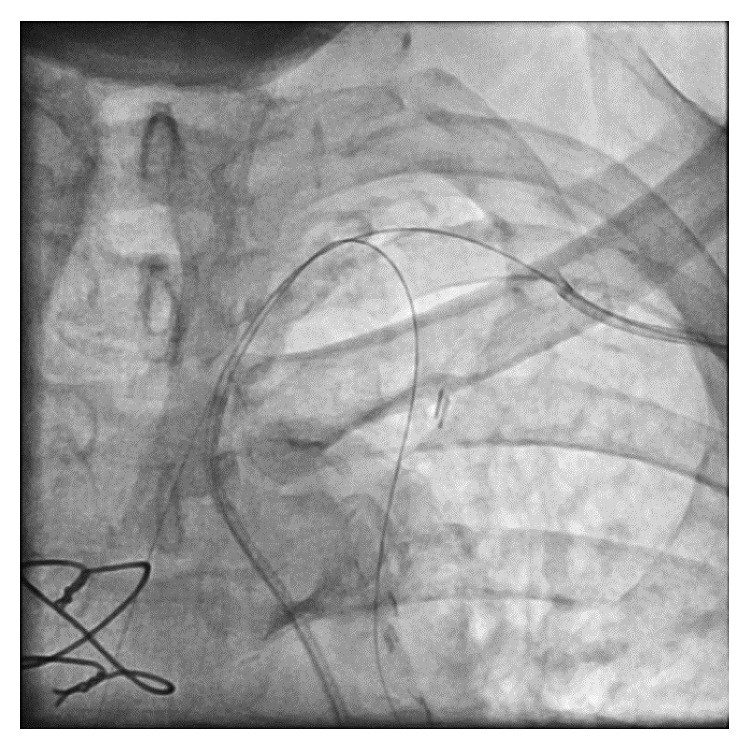
Retrograde wiring of the SA via the left radial artery and anterograde wiring of the LIMA via the left femoral artery. SA, subclavian artery; LIMA, left internal mammary artery.

**Figure 3:**
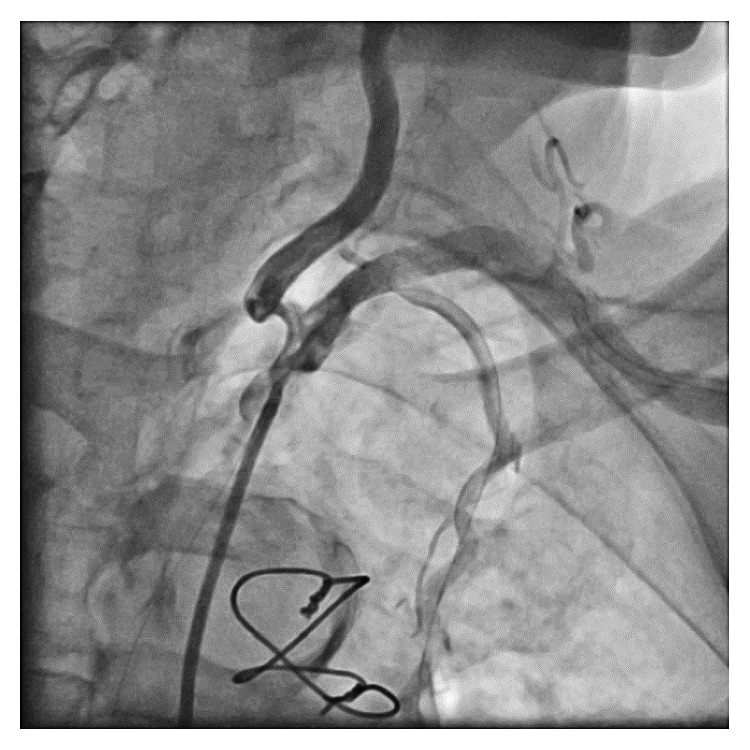
SA stented, trapping the LIMA wire. Persistent significant disease is noted in the proximal LIMA. SA, subclavian artery; LIMA, left internal mammary artery.

A 6 Fr FR 4.0 Medtronic Launcher coronary guide catheter was placed via the left femoral artery (LFA) into the proximal SA, and a 6-French IMA Medtronic Launcher coronary guide catheter was placed via the left radial artery (LRA) into the SA distal to the LIMA origin. Initial attempts to cross the diffusely calcific SA stenosis anterograde via the LFA, using multiple wire sizes and tips, were unsuccessful. A 0.018 Terumo Glidewire Advantage wire was then placed retrograde via the LRA across the SA stenosis and into the ascending aorta. A 0.014 Terumo Runthrough NS Hypercoat coronary guidewire was placed from the LFA into the LIMA (Figure 2). The SA was predilated via the LRA. A 5 mm × 28 mm balloon expandable stent (Multi-Link Ultra, Abbott Vascular) was then placed into the SA crossing the ostium of the LIMA, trapping the LIMA guidewire (Figure 3). This was followed by postdilatation of the stent with a 5 mm × 15 mm noncompliant balloon (NC Trek, Abbott Vascular). A second Terumo Runthrough NS Hypercoat coronary guidewire was then placed through the stent into the LIMA from the LFA, and the trapped guidewire was removed. Multiple unsuccessful attempts were made to pass a balloon through the stent struts into the LIMA from the LFA. Despite the use of small, low profile balloons and a guide extender (Guide Liner, Vascular Solutions), the LIMA could not be accessed from the LFA. At this point, the decision was made to change the angle of approach to the LIMA by attempting percutaneous intervention via the LRA.

A 0.014 Terumo Runthrough NS Hypercoat coronary guidewire was then placed from the LRA through the stent struts into the LIMA. The coronary guidewire which had been placed into the LIMA from the LFA was removed. A 0.035 Terumo Glidewire Advantage wire was then placed from the LFA into the SA.

**Figure 4:**
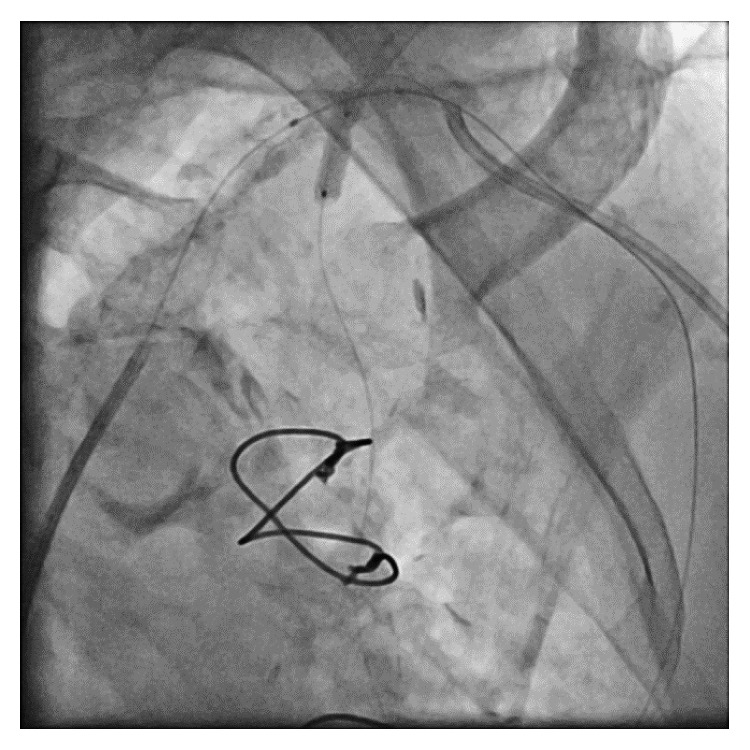
The DES is deployed in the LIMA with a balloon placed in the SA. The balloon will be inflated following stent deployment prior to concluding with final kissing angioplasty. DES, drug-eluting stent; LIMA, left internal mammary artery; SA, subclavian artery.

**Figure 5:**
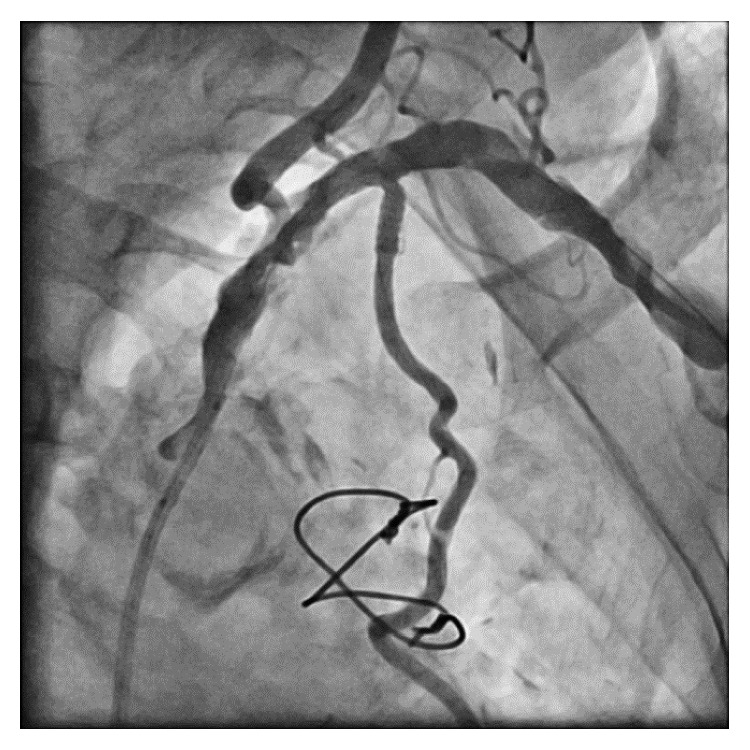
Final angiogram demonstrating a patent SA/LIMA bifurcation. SA, subclavian artery; LIMA, left internal mammary artery.

The stent struts were then dilated via the LRA, and a 3 mm × 12 mm drug-eluting stent (Xience Alpine, Abbott Vascular) was easily passed through. A 5 mm × 15 mm noncompliant balloon (NC Trek, Abbott Vascular) was then placed into the SA stent via the LFA, and a small portion of the LIMA stent was pulled back into the SA stent in order to perform “T and small Protrusion” or TAP stenting [4] of the SA/LIMA bifurcation (Figure 4). A final kissing balloon angioplasty was accomplished, and post-op angiography demonstrated a patent SA/LIMA bifurcation (Figure 5).

The patient was symptom free with improved LV ejection fraction to 30% by echocardiography at 6 months.

## 3. Discussion

Subclavian artery stenosis (SAS) is found in up to 18% of patients with PAD [5]. Proximal left SAS was found to occur in 11.8% of patients with both PAD and coronary artery disease requiring CABG [2, 6]. In patients who have undergone CABG with LIMA, the estimated incidence of CSSS is between 0.2% and 6.8% [2, 7]. Based on data from 98 case reports, the most common manifestation of CSSS is stable/unstable angina (88.6%), MI (11.4%), and CHF or worsening LV systolic dysfunction (13.2%) [8].

Endovascular treatment of SAS has evolved into first-line therapy. Balloon expandable stents have been associated with low periprocedural complication rates. In addition, ten-year patency and event-free survival appear comparable to surgical treatment of SAS [2, 9].

The case described is anatomically unique due to the presence of extensive atherosclerosis of the subclavian/LIMA bifurcation resulting in severe ischemia leading to progressive LV systolic dysfunction and congestive heart failure. This complex bifurcation anatomy creates not only substrate for CSSS but compromises inflow to and outflow from the LIMA, likely compounding the severity of the LAD territory ischemia causing significant cardiac compromise [10].

**Figure 6:**
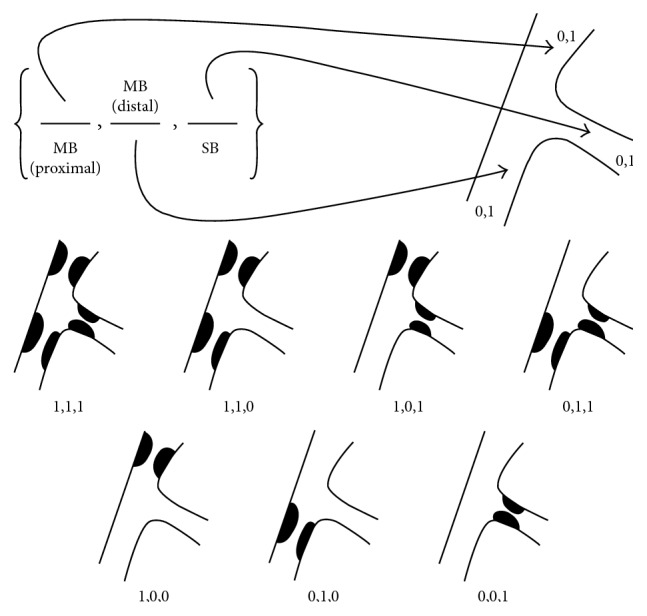
The Medina classification of coronary bifurcation lesions [11].

The lesion was classified as a Medina 1,1,1 bifurcation type (Figure 6) using the Medina classification for coronary bifurcations [11]. In this case, the SA was considered the main branch (MB) and the LIMA the side branch (SB), and the procedure was planned accordingly.

Accessing the lesion from both the proximal and distal SA (MB) provided a unique approach to bifurcation stenting, which is not available in the coronary tree. The decision to utilize a two-stent technique was based on the large size of the SB (LIMA) with disease extending greater than 5 mm from the carina into the SB [12]. The “T and small protrusion” or TAP technique was the chosen bifurcation technique [4]. The “crush” bifurcation technique has been reported in SA/vertebral artery bifurcation stenoses [13]; however, the technically less demanding TAP technique was chosen based on lesion complexity and difficult lesion access [4]. Also, the near ninety-degree angle of the bifurcation favors the TAP technique [12]. It should be noted that despite the high-risk nature of the intervention, mechanical support was not utilized. This patient had significant bilateral lower extremity PAD, including a totally occluded right iliac artery making the use of support devices problematic. This case illustrates a severe clinical manifestation of CSSS and LIMA compromise secondary to a complex bifurcation stenosis. As demonstrated, with careful planning and the meticulous use of bifurcation techniques adapted from the coronary experience, a procedure of this nature can be performed safely and with an excellent outcome.
